# Editorial: Reviews in reproductive epidemiology: 2022

**DOI:** 10.3389/frph.2023.1287513

**Published:** 2023-11-07

**Authors:** Stefania Papatheodorou, Vassiliki Benetou

**Affiliations:** ^1^ School of Public Health, Harvard University, Boston, MA, United States; ^2^ Department of Hygiene, Epidemiology and Medical Statistics, School of Medicine, National and Kapodistrian University of Athens, Greece

**Keywords:** Human Papilloma Virus (HPV), male infertility, health promotion, personhood, bacterial vaginosis (BV), review

**Editorial on the Research Topic** Reviews in reproductive epidemiology: 2022

Under the current landscape of the exponential increase in primary studies of all study designs in reproductive epidemiology on a wide range of reproductive health issues as well as the changing access to reproductive health services, summarizing and interpreting current evidence is of utmost importance for clinicians, policy makers, public health officials and individuals. This special collection of *Frontiers in Reproductive Health* under the Research Topic *Reviews in Reproductive Epidemiology* aimed to bring together the most recent evidence synthesis on pressing topics of reproductive epidemiology, to highlight current advances or gaps in knowledge and to propose new associations and/or effective interventions. Summarizing all available evidence in a topic highlights the methodological differences between studies that answer the same question and incorporate quality considerations in the interpretation of the totality of evidence.

This Research Topic consists of four reviews covering topics such as HPV vaccination, attributes of personhood, bacterial vaginosis treatments and male infertility. Krokidi et al. summarized available evidence on an important issue related to the effectiveness of health education interventions about uptake, acceptance and awareness on Human Papilloma Virus (HPV) vaccination among people 9–29 years old in India, in light of the new indigenous HPV vaccine (Krokidi et al.). In their systematic review, after searching three databases up to July 2022, seven studies conducted in India were included. Authors concluded that health education and promotion interventions such as audiovisual presentations and workshops proved to be effective in increasing uptake, awareness, and acceptance of the HPV vaccine, while the barriers included, among others, the cost of the vaccine, the lack of awareness, and cultural issues. Another noteworthy conclusion was that males and marginalized populations were underrepresented whereas the involvement of various stakeholders was proven beneficial, and it is highly recommended.

In a special and very interesting assessment, Hughes and Hughes proposed a framework, based on the biological discrete events and processes spanning pre-fertilization and prenatal development, which implies that personhood should be incrementally attributed, and societal protections should be graduated and applied progressively across the pre-birth timespan (Hughes and Hughes). They provide a novel perspective for considering the biological and ethical aspects of attribution of personhood and individualization of potential humans.

In a comprehensive assessment, Abbe and Mitchell systematically appraised ongoing and potential treatment and prevention strategies for bacterial vaginosis (BV) (Abbe and Mitchell). BV, which is a common cause of vaginitis worldwide, is associated with serious adverse reproductive outcomes including preterm birth, sexually transmitted infections and pelvic inflammatory disease. Given that antibiotics (such as metronidazole and clindamycin) are the only FDA approved treatment and that 50%–80% of women experience recurrence within a year of completing the antibiotic treatment, implementation of new strategies for treatment and prevention is crucial. Thus, the review presents current options of BV management such as smoking cessation, condom use and hormonal contraception, additional strategies considered by many people such as dietary modifications, non-medical vaginally applied products, and treatments from medical practices outside of allopathic medicine. Also, other less studied but promising treatments are presented including probiotics, vaginal microbiome transplantation, pH modulation, and biofilm disruption.

Finally, Kaltsas et al. tried to answer the question whether varicoceles in men with non-obstructive azoospermia (NOA) should be operated or not (Kaltsas et al.). The prevalence of this condition in the general population is 1% while it can be as high as 15% in the population of infertile men. Among non-obstructed azoospermic men, 4%–14% have varicocele. Several studies have been performed to assess whether varicocele repair may contribute to the reappearance of spermatozoa in semen. This hypothesis is also contradicted by the small number of spontaneous pregnancies after such interventions. The findings of this review conclude that varicocelectomy in NOA-men may have a beneficial effect on spermatogenesis and the reappearance of motile spermatozoa in the ejaculate. In addition, varicocelectomy increases sperm retrieval rate in men who remain azoospermic after the surgery. However, there is a possibility that NOA-men who are positive for spermatozoa in the semen samples post-varicocele repair will relapse into azoospermia. As a result, NOA-men should be advised to freeze spermatozoa appearing the semen post-varicocelectomy. Finally, this review highlighted that the performance of varicocelectomy in NOA-men and subsequent ICSI procedures using testicular spermatozoa may increase pregnancy and live birth rates in couples without female infertility factors.

Overall, the four contributions published in this Research Topic provide valuable information on the timely topics of HPV vaccination, origins of personhood, sexually transmitted diseases and male infertility. Given the representation of broad reproductive epidemiology research areas, this Research Topic highlights recent advances of the distinct characteristics of each topic, whilst emphasizing important directions and new possibilities for future inquiries. The Research Topic also highlights the limitations that the authors come across when aiming to combine studies with different designs and potential biases and how these considerations were incorporated in the critical appraisal of evidence synthesis.

